# Effect of CNT Contents on the Microstructure and Properties of CNT/TiMg Composites

**DOI:** 10.3390/ma12101620

**Published:** 2019-05-17

**Authors:** Xiaomin Yuan, Haonan Zhu, Huiling Ji, Yiwei Zhang

**Affiliations:** 1 School of Materials Science and Engineering, Anhui University of Technology, Ma’anshan 243002, China; zhuhaonanslzm@163.com; 2 Department of Mechanical Engineering, Industrial & Commercial of Anhui University of Technology, Dangtu 243100, China; yw2hl@126.com

**Keywords:** carbon nanotubes, MgH_2_, hot pressing sintering, Ti-Mg alloy

## Abstract

Carbon nanotubes (CNTs), dispersed in absolute ethanol, were evenly mixed into Ti/MgH_2_ powders by wet milling. Then, we applied the vacuum hot-pressed sinteringmethod to the CNTs/TiMg composite materials. An optical microscope (OM), X-ray diffraction (XRD), scanning electron microscopy (SEM) with energy dispersive spectroscopy (EDS) and a field emission scanning electron microscope (FESEM) were used for the microstructure observation and phase analysis of samples. The mechanical properties were measured via the micro-vickers hardness. The results show that the main phases in the composites were Ti, Mg and C. Meanwhile, a small amount of Ti-Mg solid solution phase was also found. The cross-section morphology of the composites shows that the melted magnesium fills the grain interface during extrusion and that the composites have a better compactness.The microstructures of the composites have been greatly refined as the CNT contents increased. The structure of the composites was further refined when 0.5 wt.% CNTs were added. The fracture surface is obviously a ductile fracture. The microhardness increases obviously with the CNT content increasing. When the content of the CNTs is 1.0 wt.%, the microhardness of the composites reaches 232 HV, which is 24% higher than that of the matrix.

## 1. Introduction

Since the new century, people have increased the demand for light-weight and high-strength materials with the development of the society. With the increase of material strength, the size and quality of materials required for some load-bearing structures also decreases. In the field of aircraft and automobiles, this will bring some advantages, such as the increase of the payload and fuel efficiency [1]. With the decline of global oil resources, improving the fuel efficiency of engines has become a great expectation. Metals and alloys do not provide enough strength and stiffness for the structure. This has led to the development of metal matrix composites (MMCs). Therefore, the metal matrix provides toughness and ductility. Strength and stiffness are provided by ceramic or high hardness metal-based particles or fiber reinforced materials.

Compared with other reinforcements, carbon nanotubes (CNTs) have the advantages of a low density and high modulus. Not only can they lighten the weight of composites, but they can also improve the mechanical properties of composites. They are the best reinforcement of composites. At present, CNTs have been widely used in metals, ceramics, polymer and cement. Many studies have shown thatCNTscan be used as reinforcing materials in order to achieve a great reinforcing effect [2,3,4]. Meanwhile, titanium is the preferred material to develop structural parts with a lightweight and high performance. This is because it has a low specific gravity, high strength and great corrosion resistance. It is widely used in aviation, navigation, petroleum, the chemical industry, biology, medicine and other industries [5]. The results show that CNT-reinforced titanium matrix composites have a good specific strength, high temperature stability and good thermal conductivity [6].

Moreover, CNTs have poor wettability with most metals. They do not easily bond with the metal matrix to form a solid interface. Then, CNTs can not fully perform their reinforcing effects. This reduces the properties of CNT-reinforced metal matrix composites in all aspects. At present, there are two methods to improve the wettability between CNTs and the metal matrix. One way is to modify CNTs. Another is to coat CNTs on the metal layer of the matrix or to form amphoteric carbides on the interface. However, the preparation processes are complex and expensive [7,8,9,10]. Adding an Mg element (using Mg or MgH_2_ as the activator) without changing the preparation process can improve the wettability of CNTs and matrix metals [11]. Thus, the enhancement effect of CNTs can be improved. Compared with other methods mentioned above, this method has the advantages of a simple process and low cost [12]. It has great significance for future application [13]. In this paper, MgH_2_, with a higher activity, will be added to form the composite materials. Active magnesium and hydrogen will be decomposed from MgH_2_ during hot pressing sintering. This will improve the sintering properties of the composites. It can also improve the wettability of CNTs and the Ti matrix. Meanwhile, this way can improve the interface bonding properties between CNTs and alloys. The Mg element can also play a role in refining the grain size [14].

Many studies have shown that the content of CNTs has a great influence on the properties of composites [15,16,17]. CNTs have a very small volume and size, large aspect ratio and specific surface area. There are large van der Waals forces and strong electrostatic interactions between CNTs. Therefore, CNTs are very prone to entanglement or reunion in their application [18,19]. Therefore, a proper amount of CNTs can greatly improve the properties of composites. Excessive CNTs will reduce the properties of composites [20]. In this paper, the effect of CNT contents on the properties of CNT/TiMg composites will be discussed.

## 2. Experimental Section

### 2.1. Materials

The purity of the titanium powder used in this experiment is 99.1%. It containsvery small amounts of C, O and Fe elements. The composition of the MgH_2_ powder is 95% MgH_2_ and 5% Mg. The particle sizes of both powders are 300 meshes. MultiwalledCNTs are used as reinforcing materials.

### 2.2. Sample Preparation

**Figure 1:**
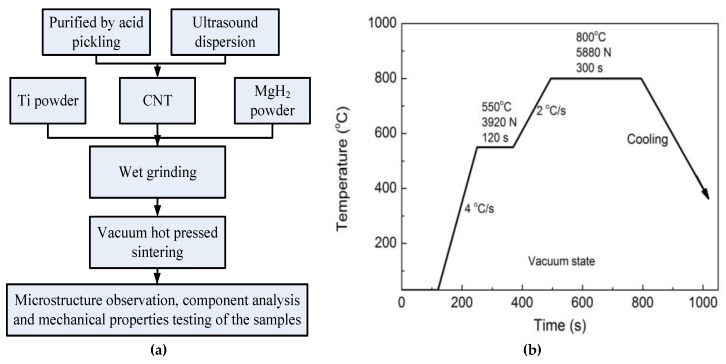
(**a**) The process flow chart and (**b**) the sintering process of the composites.

The original multi-walled carbon nanotubes will be purified by acid pickling in aqua regia. The titanium powder and magnesium hydride powder were weighed according to the mass ratio of 80:20. The CNTs with a total mass of 0 wt.%, 0.5 wt.% and 1.0 wt.%, respectively, were placed in anhydrous ethanol and dispersed uniformly by ultrasonic wave. Then, the metal powders were mixed with CNT dispersions. They will be manually grinded for 2 h by a wet method to obtain three kinds of powders. After drying, the powder is loaded into a graphite mold. The mixed powder is separated from the upper and lower die heads and the inner cavity of the graphite die by graphite paper. Then, the three kinds of powders were sintered by hot pressing at the same sintering temperature (800 °C) and holding time (15 min). Vacuum hot-pressing sintering was carried out with a Gleeble 3500 thermal simulator (DSI, New York, NY, USA). The technological parameters of hot pressing sintering are as follows: after the vacuum evacuation, the temperature is first raised to 550 °C at a heating rate of 4 °C/s for 2 min, and then the powders are pressed to 3920 N at a heating rate of 2 °C/s, then pressed to 800 °C at the sintering temperature, and kept under 5880 N pressure for 5 min, cooled to room temperature, after which the film is removed. Figure 1 is the process flow chart and sintering process. After the composites were prepared, the microstructures and properties of the samples were observed and tested respectively.

### 2.3. Characterization 

The effects of the carbon nanotube content on the CNTs/titanium-magnesium composites were investigated by testing and analyzing the micro-morphology, cross-section composition and microhardness of the surface and fracture surface of the samples. The samples were prepared metallographically in a conventional manner, and ground on successively finer silicon papers from 200 to 1000 grade. The samples were polished on cloths with a 2.5 µm diamond paste and water-based lubricant. The samples were put into anhydrous ethanol, cleaned and dried by ultrasonic wave. The surface and cross section of the samples were observed by JSM-6490LV type (JEOL, Tokyo, Japan) scanning electron microscopy (SEM, 6490LV, JEOL, Tokyo, Japan) with backscattered electron, and the elements of the samples were analyzed by energy dispersive spectroscopy (EDS) (X-Max, Oxford, London, UK). The fracture samples were measured by Nano SEM 430 type (FEI, Hillsboro, OR, USA) field emission scanning electron microscopy (FESEM). Then, the composites were analyzed by a D8 Advance type polycrystalline X-ray diffractometer (Bruker, AXS, Karlsruhe, Germany). The scanning angle was 20–80 degrees. Furthermore, the scanning speed was 0.5 degree/s. After the grinding and polishing, the hardness of the specimens was respectively measured at one quarter of the longitudinal section and at the center of the specimens. Each value has five groups that were measured, and the average values were obtained. The hardness was measured by HV-1000 type micro-hardness tester (Weiyi Testing Instrument Co., Ltd., Laizhou, China). The loading load was 4.903 N, and the holding time was 10 s.

## 3. Results and Discussion

**Figure 2:**
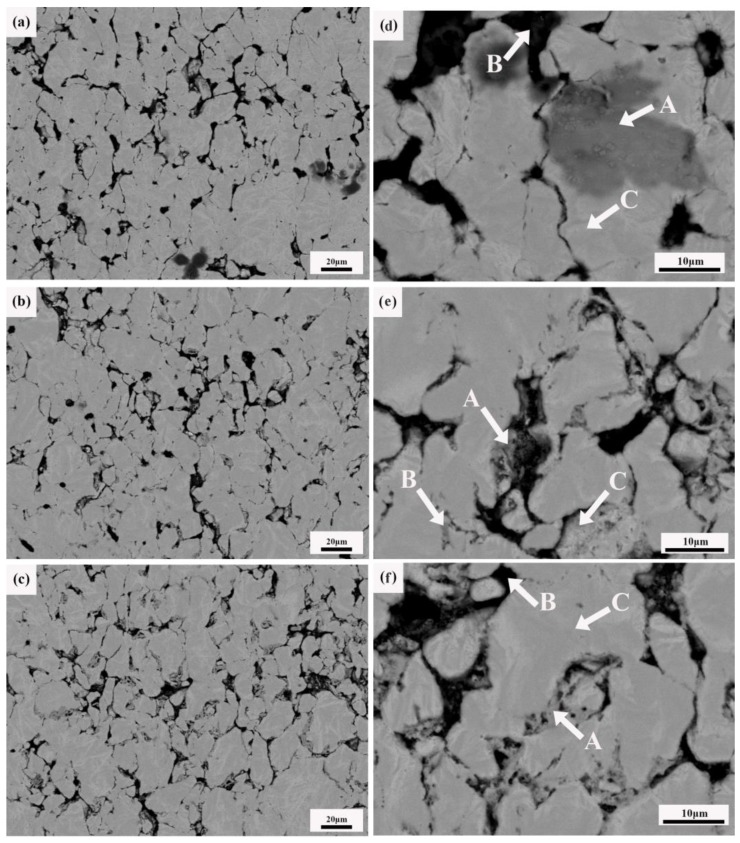
The SEM morphology and EDS analysis results of composites with different CNT contents (**a**,**d**) 0%; (**b**,**e**) 0.5%; and (**c**,**f**) 1.0%.

Figure 2 shows the SEM morphology and EDS analysis results of the composites with different CNTcontents. Furthermore, the results of the EDS analysis are shown in Table 1.

As shown in Figure 2a,d, it can be seen that there is a white massive structure, black gap and a few gray microstructures on the cross-section of the composites. According to the results of the energy spectrum analysis, the composition of the white massive microstructure is pure titanium, and there are two elements, Ti and Mg, in the black gap and in the gray structure. The black gap contains the most magnesium. The gray microstructure should be composed of a small amount of solid solution of magnesium and titanium.

Figure 2b,e shows the cross-sectional morphology and energy spectrum analysis of the composite sample containing 0.5 wt.% CNTs. Compared with Figure 2a,d, the size of the composites’ microstructure decreases, and the uniformity increases. The black gap decreases, but the number increases, and the distribution becomes more uniform. At the same time, the energy spectrum analysis showed that the composition of the white massive microstructure was all Ti, and there were two elements, Ti and Mg, in the black gap and gray microstructure. The Mg element is distributed at the grain boundary and interstice of the Ti structure. It can be found that although the Mg element exists at the grain boundary, the content of Ti is still much higher than that of Mg.

Figure 2c,f shows the cross-sectional morphology and energy spectrum analysis of the composite sample containing 1.0 wt.% CNTs. Compared with the former, the size of the black gap is significantly reduced. The gray microstructureis dispersed more evenly, and the white massive microstructure did not change significantly. However, the energy spectrum showed that the content of Ti in the black gap increased. At the same time, there is a small amount of magnesium in the white microstructure. With the increase of the CNT contents, the grey structure increases obviously and disperses more evenly in the composites. At the same time, the number of gaps decreases, and the size of the gaps decreases markedly. The results show that the proper increase of the carbon nanotube content can dissolve magnesium into titanium and refine the grain better.

In summary, the addition of CNTs during sintering provides a lot of non-uniform nucleation centers for melted Mg. Mg is more refined. Melted Mg flows between the Ti particles to form composite materials. However, due to the influence of the sintering parameters such as thedwell time being short, the fluidity of Mg is poor, resulting in an uneven distribution of Mg. Consequently, there are still gaps in the microstructure of the composite.

**Figure 3:**
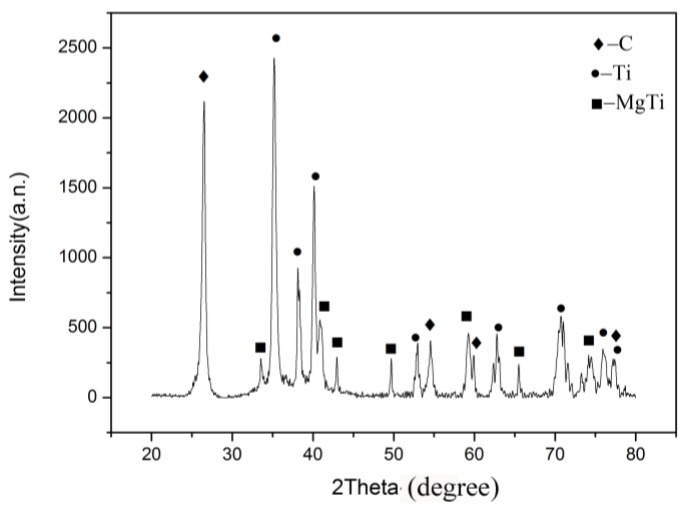
The XRD spectrum of the composites with 1.0 wt.% CNTs.

The XRD diagram of the CNT composites with 1.0 wt.% content showing the diffraction peak of the Ti-Mg solid solution phase appears in Figure 3. The carbon diffraction peak is graphite rather than CNTs in the XRD spectrum. This was because the sintering mold is made of graphite. When the sample is hot pressed and sintered during the sample preparation, the composite markedly shows carbon in the samples. Meanwhile, the CNT powders are amorphous, and the diffraction peak is a broad diffraction peak like steamed bread in the literature [21]. It was found that the main factors affecting the reinforcement properties of CNTs are an undispersed uniformity and the reaction with the metal matrix. The CNTs will react easily with titanium to form the TiC phase at a high temperature. However, the XRD analysis showed that there is no TiC phase. It was shown that the sintering temperature is lower than the reaction temperature of the CNT and Ti powders. Meanwhile, it was proven that the CNTs were not damaged in large quantities. This shows that there is no TiC phase in the composites. The CNTs have not been damaged in large quantities. Consequently, carbon nanotubes can play a certain role in the strengthening process. At the same time, Ti and CNTs formed Ti-Mg solid solution new phases during the hot-pressing sintering. With the addition of MgH_2_, activated magnesium dissolves into titanium grains. The solid solution of magnesium and titanium formed, and meanwhile the grains were refined.

**Figure 4:**
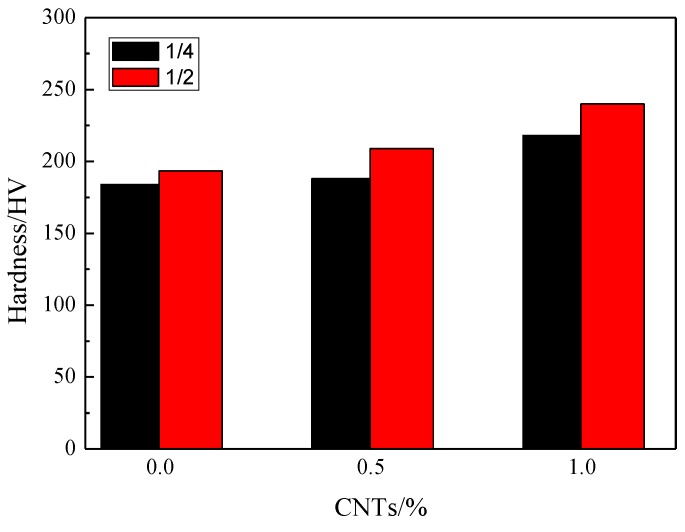
The microhardness of the composites with different CNT contents.

Figure 4 shows the microhardness changes of the composites of different positions with different CNT contents. Table 2 shows the results of the microhardness test of the CNT/TiMg composites with different CNT contents at different cross sections and thicknesses. The hardness of the quarter of the sample was observed. The microhardness of the Ti/Mg composites without the CNTs is 184 HV. With the CNT contents increasing, the microhardness increased from 188 HV to 218 HV. Observed for half of the samples, the microhardness of the Ti/Mg composites without the CNTs is 187 HV, with 0.5 wt.% CNTs it is 202 HV and with 1.0 wt.% CNTs it is 232 HV. The experimental data shows that the hardness of the composites increases markedly with the increase of the CNT contents. The hardness at one-half of its thickness is higher than that at one-quarter of its thickness. This was because MgH_2_ will be decomposed into magnesium and hydrogen at a high temperature. At the same time, hydrogen will diffuse outward under pressure during the forming process. Therefore, the center of the composites will be denser than one quarter of the composites. The pores and defects are present in less than one quarter of the longitudinal section in the composites. This showed that the CNT contents have an effect on the mechanical properties. 

In conclusion, CNTs can improve the mechanical properties of the composites to a certain extent as a reinforcing phase of metal matrix composites. CNTs can also make the Ti-Mg alloy bond more closely. The strengthening reasons are as follows. (1) CNTs with excellent mechanical properties share part of the external loads, so that the loads shared by the matrix can be reduced and not easily destroyed. (2) CNTs can promote the formation of a Ti-Mg solid solution. CNTs contribute in refining the grain and improving the properties of composites. (3) After adding CNTs, because of the difference of physical and chemical properties between CNTs and the metal matrix, the dislocation density of the composites increases. There are many strengthening mechanisms of metal materials, such as solution strengthening, second phase strengthening, dislocation strengthening, fine grain strengthening and precipitation strengthening. When there are dislocations in the crystal, the complete ordered structure of the crystal will be destroyed. The lattice distorts and the storage energy increased. The resistance to plastic deformation will be strong. Generally, if the dislocation density is higher, there is more energy inside the material. The effect of the dislocations on hindering the slip becomes great, and then the composites can exhibit a better strength. The dislocation motion will be hindered by external forces. The hardness of CNTs increases by 8% compared with the matrix when the content of CNTs is 0–0.5 wt.%. The hardness increases by 24% when the content of CNTs is 0.5 wt.%–1.0 wt.%. The hardness increases more obviously when the content of CNTs is between 0.5 wt.% and 1.0 wt.%. The results show that the higher the CNT content, the harder the material becomes when the CNTcontents are under 1 wt.%.

**Figure 5:**
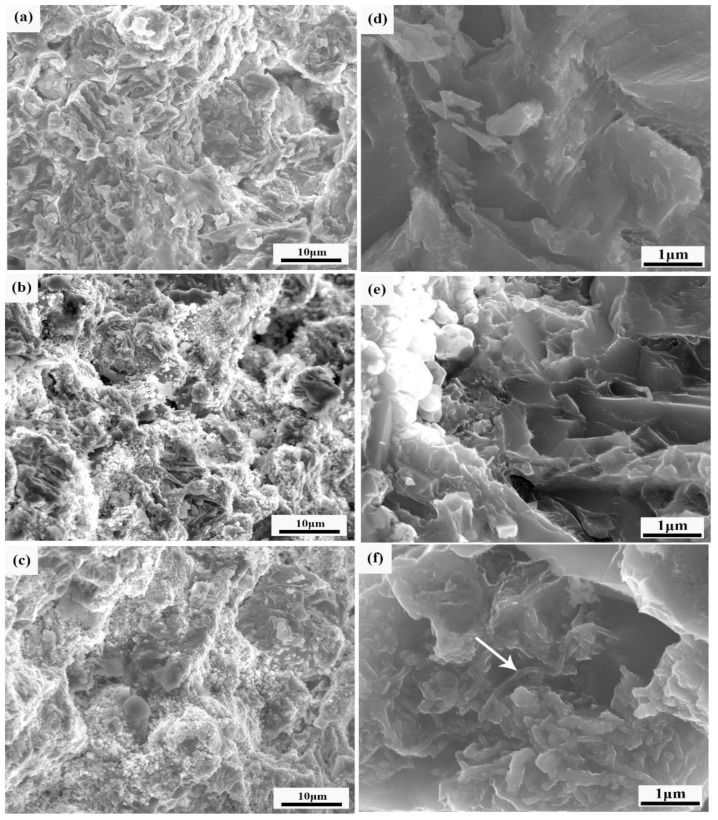
FESEMfracture morphology of composites with different CNT contents (**a**,**d**) 0%; (**b**,**e**) 0.5%; and (**c**,**f**) 1.0%.

Figure 5 is the fracture morphology of the CNT-reinforced titanium-magnesium composites. As can be seen from the graph, the fracture morphology of CNTs varies greatly from low to high. The composites with 0 wt.% CNTs have a smooth fracture surface and obvious layered massive structure, which belongs to the brittle fracture. When the content of the CNTs increased, there were many dimples and tearing edges at the fracture surface, which showed a ductile fracture. It can be seen from Figure 5c that the CNTs are well dispersed in the matrix, and no large area of CNT agglomeration can be observed. In Figure 5f, the arrow points to the position where you can clearly discover the existence of the CNTs. This is consistent with the results of the XRD analysis. CNTs embedded in the metal matrix play a reinforcing role for composite materials. Because of the excellent properties of CNTs, CNTs embedded in the matrix can improve the mechanical properties of sintered bulk samples. At the same time, the addition of CNTs is conducive to the formation of a fine Ti-Mg solid solution structure, which can play the role of grain refinement [22].

From the above analysis, it can be seen that a proper amount of CNTs as a reinforcing phase of metal matrix composites does play a significant positive role in improving the toughness of matrix materials. However, attention should be paid to controlling the content of CNTs and ensuring that they are evenly distributed in the matrix, so as to avoid a large number of agglomerations. This can prevent the formation of a large number of weak phases that reduce the properties of composites. At the same time, the influence of the carbon nanotube content on composites is also related to the dispersion of CNTs in the composites.

## 4. Conclusions

In this paper, there are several conclusions that are obtained, based on the results provided by the X-ray diffraction, SEM techniques and micro-hardness used in order to investigate the microstructure evolution of the alloys and mechanical properties in the present work. The following conclusions can be drawn according to the research in this paper:

1. The microscopic morphology and energy spectrum analysis show that, with the increase of the carbon nanotube content, the number of pores on the surface of the composite increases, but the size decreases. Meanwhile, the structure of the composites is finer and more uniform after adding CNTs.
2. The XRD analysis of the composites shows that with the increase of the CNT contents, new phases appear in the composites, and a Ti-Mg solid solution phase is formed, which indicates that CNTs can promote the diffusion of Mg and the solid solution of Mg into Ti.
3. The fracture analysis of the composites shows that when CNTs are not added, larger particles can be observed at the fracture surface, which is a quasi-cleavage fracture. A fine structure and dimples can be observed at the fracture surface after adding CNTs, which is a ductile fracture. Therefore, CNTs and the matrix form an effective bond.
4. The microhardness analysis of the composites shows that the microhardness of the composites increases with the increase of the carbon nanotube content. When 1 wt.% CNTs is added, the microhardness of the composites reaches the highest at 232 HV. Compared with the composite without CNTs, the microhardness of the composite increased by 24%.

## Figures and Tables

**Table 1:** Energy spectrum analysis of the composite section.

	Position	Mg-K (wt.%)	Ti-K (wt.%)	C-K (wt.%)
Figure 2d	A	9.26	90.74	
	B	15.75	84.25	
	C		100	
Figure 2e	A	4.56	95.44	
	B	3.12	96.88	
	C		100	
Figure 2f	A	2.76	97.24	
	B	0.79	5.86	93.35
	C	1.42	98.58	

**Table 2:** Microhardness of the composites with different CNT contents.

CNT Contents (wt.%)	Thickness Location of Longitudinal Section	Micro-Hardness (HV)					Average Value (HV)
0	1/4	203	174	183	166	193	184
	1/2	200	245	160	155	173	187
0.5	1/4	170	187	194	214	175	188
	1/2	201	213	214	207	174	202
1.0	1/4	225	227	214	205	217	218
	1/2	231	212	242	254	218	232
